# A transient receptor potential channel-related model based on machine learning for evaluating tumor microenvironment and immunotherapeutic strategies in acute myeloid leukemia

**DOI:** 10.3389/fimmu.2022.1040661

**Published:** 2022-12-16

**Authors:** Jingsheng Hua, Tianling Ding, Yanping Shao

**Affiliations:** ^1^ Department of Hematology, Taizhou Municipal Hospital, Taizhou, China; ^2^ Department of Hematology, Huashan Hospital, Fudan University, Shanghai, China; ^3^ Department of Hematology, Taizhou Hospital of Zhejiang Province affiliated to Wenzhou Medical University, Taizhou, China

**Keywords:** acute myeloid leukemia, TRP, signature, schip1, machine learning

## Abstract

**Background:**

Acute myeloid leukemia (AML) is an aggressive hematopoietic malignancy. Transient receptor potential (TRP) channels in AML still need to be further explored. A TRP channel-related model based on machine learning was established in this study.

**Methods:**

The data were downloaded from TCGA-LAML and Genome-Tissue Expression (GTEx). TRP-related genes (TRGs) were extracted from previous literature. With the use of Single-Sample Gene Set Enrichment Analysis (ssGSEA), TRP enrichment scores (TESs) were calculated. The limma package was used to identify differentially expressed genes (DEGs), and univariate Cox regression analysis was performed to identify prognostic DEGs. The above prognostic DEGs were analyzed by Random Survival Forest and least absolute shrinkage and selection operator (Lasso) analysis to create the TRP signature. The Kaplan–Meier and receiver operating characteristic (ROC) curves were plotted to investigate the efficiency and accuracy of prognostic prediction. Moreover, genomic mutation analysis was based on GISTIC analysis. Based on ESTIMATE, TIMER, MCPcounter, and ssGSEA, the tumor microenvironment and immunological characteristics were expressly evaluated to explore immunotherapeutic strategies. Enrichment analysis for TRP signature was based on the Kyoto Encyclopedia of Genes Genomes (KEGG), Gene Ontology (GO), over-representation analysis (ORA), and Gene Set Enrichment Analysis (GSEA). Genomics of Drug Sensitivity in Cancer (GDSC) and pRRophetic were used to carry out drug sensitivity analysis. Conclusively, SCHIP1 was randomly selected to perform *in vitro* cyto-functional experiments.

**Results:**

The worse clinical outcomes of patients with higher TESs were observed. There were 107 differentially expressed TRGs identified. Our data revealed 57 prognostic TRGs. Eight TRGs were obtained to establish the prognostic TRP signature, and the worse clinical outcomes of patients with higher TRP scores were found. The efficiency and accuracy of TRP signature in predicting prognosis were confirmed by ROC curves and five external validation datasets. Our data revealed that the mutation rates of DNMT3A, IDH2, MUC16, and TTN were relatively high. The level of infiltrating immune cell populations, stromal, immune, and ESTIMATE scores increased as the TRP scores increased. Nevertheless, AML patients with lower TRP scores exhibited more tumor purity. The TRP scores were found to be correlated with immunomodulators and immune checkpoints, thus revealing immune characteristics and immunotherapeutic strategies. The IC50 values of six chemotherapeutics were lower in the high TRP score (HTS) group. Finally, it was found that SCHIP1 may be the oncogenic gene.

**Conclusion:**

The results of this study will help in understanding the role of TRP and SCHIP1 in the prognosis and development of AML.

## Introduction

Acute myeloid leukemia (AML) is an aggressive hematopoietic malignancy caused by the malignant transformation of hematopoietic stem cells or progenitor cells, which is highly heterogeneous (1). It is the most common acute leukemia in adults, with an annual incidence of approximately four per 100,000 cases (1). Patients with AML generally have a poor prognosis (1). Therefore, it is of great significance for clinical treatment to find molecular markers that can judge the prognosis and effectively distinguish whether patients can benefit from treatment. Advances in genomics have greatly improved our understanding of the pathogenesis of AML, which is one of the targets in the search for diagnosis and treatment of AML.

The prognosis of the same AML type may be very heterogeneous. Therefore, it is of great importance to evaluate the characteristics of each AML patient. In the past 40 years, many new achievements have been made in pathogenesis, but there is no innovative progress in the treatment of AML. The traditional treatment of AML mainly includes three parts: induction regimen therapy, monitoring after induction therapy, and treatment after complete remission (CR) (2). In the post-CR treatment, patients under the age of 60 should choose the appropriate treatment according to the risk stratification, indicating the important role of genetic risk stratification in guiding the treatment of AML. Traditional cytogenetic classification includes better karyotypes, intermediate karyotypes, and poor karyotypes. With the deepening of research on leukemia, people can have more profound knowledge of the pathogenesis of leukemia, and the risk classification of AML combined with genetic changes is more recommended by most guidelines (1). However, the risk classification of AML needs to be further explored.

Bioinformatics is an interdisciplinary subject involving mathematics, statistics, computer science, biology, and other disciplines. After decades of development, bioinformatics is still a subject with great development prospects (3). In recent years, a large number of databases containing biological information of various species have been established worldwide. As the largest database for cancer research, The Cancer Genome Atlas (TCGA) (4) database stores rich sequencing data and clinical information, which can be downloaded by researchers all over the world for free for research so as to improve the cognitive ability of doctors and researchers on the disease. AML project, as one of the earliest and most well-developed projects in TCGA, stores a large amount of sequencing and clinical data, which can be used to prospectively explore some disease-related information and provide directions for clinical and experimental research.

Transient receptor potential (TRP) was first discovered in *Drosophila (* 5). When *Drosophila* bearing the mutant gene is exposed for an extended time to light, its photoreceptor will show a transient increase of voltage, so it is named transient receptor potential channel (5). Mammalian TRP channels are composed of 28 cation permeation channels (6), each with six transmembrane peptides, which assemble into tetramers to form ion channels (6). TRP channels have various types, such as TRPC, TRPA, TRPM, TRPN, TRPP, and TRPV (7). TRP channels are involved in various physiological and pathological processes of the body and respond to physical or chemical stimuli in the cellular environment by sensing them (8). TRP channel is closely related to circulatory (9), urinary (10), digestive (11, 12), nervous (13, 14), and other systems. It is a cation channel widely existing in the body, mainly permeating Ca^2+^, Mg^2+^, and other cations (15). By affecting the change of cation concentration, the TRP channel changes the strength of the corresponding pathway signal in the cell, leading to the change of cell function (15). As for AML, TRP was seldom reported in AML. TRP ion channel TRPM2 could enhance the proliferation of AML cell lines through multiple pathways (16). TRP Melastatin Subfamily Member 4 may be an alternative therapeutic approach for AML (17).

Therefore, TRP may be used as a prognostic and therapeutic target. However, the TRP channel in AML still needs to be further explored.

In this study, bioinformatics analysis was used to screen out genes’ expression of AML, including TRP-related genes (TRGs) from public databases, and to analyze the characteristics of TRP enrichment in TCGA-LAML. By combining survival information and gene expression, 57 prognostic TRGs were preliminarily identified as possible AML target genes. Through the Random Survival Forest model and least absolute shrinkage and selection operator (Lasso) analysis, a predictive model consisting of eight genes was established and validated in five external datasets, thus proving good predictive ability. The risk prognosis model score was used to group the high- and low-risk groups, and it was found that the risk groups differed in immune profiles and treatment.

## Material and methods

### Data collection and preprocessing for acute myeloid leukemia

The transcriptome expression profile and corresponding clinical information of patients diagnosed with AML were downloaded from TCGA-LAML dataset in the UCSC Xena platform (https://xenabrowser.net/) (18). There were a total of 149 AML patients with corresponding data included in our study (N = 149). In addition, the transcriptome expression profile of corresponding normal control samples was downloaded from Genome-Tissue Expression (GTEx) project (https://www.gtexportal.org) (19). Meanwhile, five AML cohorts were collected, including GSE12417 (N = 79), GSE12417 (N = 163), GSE37642 (N = 136), GSE37642 (N417), and TARGET (N = 187), from Gene Expression Omnibus (GEO; https://www.ncbi.nlm.nih.gov/geo/) or Therapeutically Applicable Research to Generate Effective Treatments (TARGET; https://ocg.cancer.gov/programs/target/data-matrix) (20). The GEO data were generated from the Affymetrix (21) or Agilent (22) platform. Background correction and normalization for GEO data were carried out using Robust Multichip Average (RMA) algorithm (23). The data forms of TCGA and TARGET were transformed from fragments per kilobase of transcript per million fragments mapped (FPKM) to transcripts per kilobase million (TPM), of which the signal strength was similar to the value processed by RMA (24).

### Establishment of transient receptor potential enrichment score

The list of TRGs was extracted from the previous literature (25), which was used for enrichment score calculation. There were eight TRGs included in our study: TRPM1, TRPM2, TRPM3, TRPM4, TRPM5, TRPM6, TRPM7, and TRPM8. To identify TRP-related patterns, TRP enrichment scores (TESs) were calculated for each AML patient using Single-Sample Gene Set Enrichment Analysis (ssGSEA) algorithm (26). According to the optimal cutoff value of TESs calculated by R code (27), patients with AML were divided into the high-TES group (≥cutoff value) or low-TES group (<cutoff value).

### Establishment of transient receptor potential signature

The limma package was used to identify differentially expressed genes (DEGs) between the high-TES and low-TES groups (logFC > 1, p < 0.05) (28). Thereafter, univariate Cox regression analysis was performed to identify prognostic DEGs (p < 0.05) (29), and the Random Survival Forest model was utilized to screen out prognostic DEGs with higher importance (variable importance >0.3) based on the randomForestSRC package (30). To establish the TRP signature, the weight of regression coefficients of the prognostic genes identified by the Random Forest Algorithm was calculated using Lasso analysis (31), thus establishing the signature and computing the TRP score.

### Efficacy of transient receptor potential signature

The TRP score for 149 patients in TCGA-LAML cohort was estimated according to the method described above. The optimal cutoff was considered based on R code (27) as the threshold value to distinguish subgroups with high TRP scores (HTS) or low TRP scores (LTS). We compared survival differences between the two subgroups to assess the relationship between TRP score and overall survival (OS) by plotting Kaplan–Meier survival curves (32). Through the timeROC package, the 1-, 3-, and 5-year survival receiver operating characteristic (ROC) curves were plotted to investigate the efficiency and accuracy of prognostic prediction for the TRP score. To further verify the independence of the TRP score predicting prognosis for AML, univariate or multivariate Cox regression analyses of the TRP score and clinicopathological characteristics [age, gender, and white blood cell (WBC)] were performed.

### Genomic mutation analysis for acute myeloid leukemia with transient receptor potential score

Somatic mutation profiles of AML were obtained from cBioPortal (http://www.cbioportal.org/datasets) (33). Meanwhile, copy number variation (CNV) analysis was carried out after extracting data from FireBrowse (http://firebrowse.org/) (34). The genomic characteristics were assessed using Genomic Identification of Significant Targets in Cancer (GISTIC) analysis (35).

### Evaluation of immunological characteristics

We used the ESTIMATE (The Estimation of Stromal and Immune cells in Malignant Tumor tissues using Expression) algorithm to assess the abundance of immune cells, stromal cell infiltration level, and tumor purity and expressed them as immune score, stromal score, and ESTIMATE score, respectively (36). In addition, in order to comprehensively analyze the infiltration of immune cells in AML, we further analyzed the levels of six kinds of cells by using the TIMER 2.0 (Tumor Immune Estimation Resource 2.0) network server (http://timer.cistrome.org/) (37). We also used MCPcounter (38) and ssGSEA (26, 39) to assess the relative proportions of 10 immune cells and the infiltration levels of 28 immune cells, respectively. We extracted several immunomodulators from literature reported previously to explore the association between TRP score and immune processes (40).

### Enrichment analysis for transient receptor potential signature

Downloading from the MSigDB database, we acquired gene sets using for Kyoto Encyclopedia of Genes Genomes (KEGG) or Gene Ontology (GO) analyses (41). We implemented over-representation analysis (ORA) (42) and Gene Set Enrichment Analysis (GSEA) by using the clusterProfiler package (43).

### Drug sensitivity analysis

The Genomics of Drug Sensitivity in Cancer (GDSC) website was utilized to screen a wide range of drugs (44). The prediction model was constructed based on Ridge’s regression between drug sensitivity and expression profile of cell lines using the pRRophetic algorithm (45, 46). Subsequently, the IC50 value of corresponding chemotherapeutics for patients with AML was calculated.

### Cell culture

We randomly selected one gene, SCHIP1, from the TRP signature to perform *in vitro* cyto-functional experiments. We used one AML cell line, called K562, for *in vitro* assays. We incubated the AML cell line K562 in the incubator at an atmosphere of 37°C and 5% CO_2_ and cultured it in 90% Roswell Park Memorial Institute 1640 (RPMI 1640) medium with 10% fetal bovine serum (FBS).

### Cell transfections

To perform cell transfections, Hieff Trans™ *in vitro* siRNA Transfection Reagent supplied by Yeasen Biotechnology (Shanghai, China) was used, and the sequences of siRNA were as follows: si-NC (control group) sense (5′-UUCUUCGAACGUGUCACGUTT-3′), si-NC antisense (5′-ACG UGACACGUUCGGAGAATT-3′), si-SCHIP1 sense (5′-GGAGUCUGAAUCCUU GGAUTT-3′), and si-SCHIP1 antisense (5′-AUCCAAGGAUUCAGACUCCTT-3′). According to the kit instructions, the transfection steps were as follows: cells were collected, plates were spread on a six-well plate, and the number of cells on the transfection day was 5 × 10 (5) to 2 × 10 (6). OPTI-MEM medium, siRNA, and transfection reagent were used to prepare siRNA-PEI cationic nucleic acid transfection reagent complex and added to the cell suspension. After 4–6 h in a 5% CO_2_ incubator at 37°C, 2 ml of complete medium was added and incubated in an incubator for 72 h. The efficiency of SCHIP1 knockdown in K562 cells was confirmed by Western blotting assays.

### Western blotting assays

Protein was extracted through protein extraction reagents containing inhibitors. Ten microliters of protease inhibitor mixture, 10 µl of phenylmethylsulfonyl fluoride (PMSF), and 10 µl phosphatase mixture were added to 1 ml of the extraction reagent. The bicinchoninic acid (BCA) method was performed for protein detection: 25 µl of standard and sample to be tested was added to the microwells, 200 µl of BCA working solution was added to each well, the samples were incubated at 37°C for 30 min, and then the absorbance was detected at 562 nm on a microplate reader. TEMED containing 10% separation glue and 5% concentrate glue for gluing was successively used. After loading, it was electrophoresed with glycine buffer. After electrophoresis, a polyvinylidene difluoride (PVDF) membrane was used for the transmembrane of the gel. After the membrane transfer, the membrane was blocked with 5% non-fat milk and then washed three times with TBST. After blocking, the cells were incubated with primary antibodies at 4°C overnight. Before and after incubation with a secondary antibody for 1 h at room temperature, the membrane was washed with TBST three times. Finally, the color was defined according to the chemiluminescence kit, photos were taken, and statistics and analysis were performed on the gel imaging system.

### Cell Counting Kit-8 assays

Cells were collected at a concentration of 1 × 10 (4) cells/ml. Each well of the 96-well plate was inoculated with 100 µl of cell suspension, and each group had three wells. Ten microliters of si-SCHIP1 or si-NC was added to the corresponding wells and then placed into the incubator for routine culture. The next day, 10 µl of Cell Counting Kit-8 (CCK8) solution was added at a fixed time and incubated in the incubator for 0.5–4 h. Finally, the absorbance at 450 nm was measured by a microplate reader, and the cell viability was calculated.

### Statistical analysis

Normally distributed variables and non-normally distributed data between two groups were compared by t-test and Wilcoxon test, respectively. OS status estimated by Kaplan–Meier survival curves and Cox regression used for survival analysis were compared by the survminer package. ROC curves were plotted by the timeROC package, and heatmaps were plotted by the pheatmap package. R package ggplot2 (v4.1.2) was used to visualize the data. *In vitro* assays were performed for more than three independent experiments or replicates. p < 0.05 was considered statistically significant.

## Results

### Characteristics of transient receptor potential enrichment in TCGA-LAML

**Figure 1:**
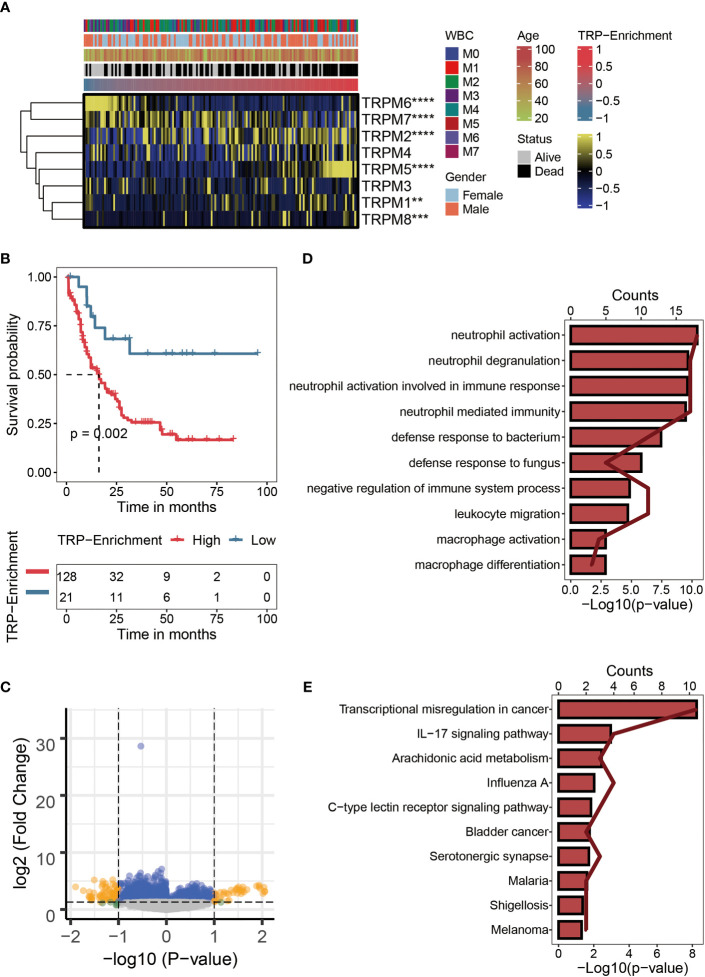
Characteristics of TRP enrichment scores in TCGA-LAML cohort. **(A)** Correlation between TRP enrichment scores and the expression values of eight TRP genes in TCGA-LAML cohort. Yellow represents high gene expression; blue represents low gene expression. **(B)** Kaplan–Meier curve showing the correlation between TRP enrichment scores and survival status of AML patients. The blue curve represents the group with lower TRP enrichment scores, and the red curve represents group with higher TRP enrichment scores. **(C)** Volcano map of differential analysis between high and low TRP enrichment groups. There were 107 differentially expressed genes between the two groups. Yellow dots indicate genes whose expression values differ between the two groups, while blue dots indicate genes whose expression values do not differ between the two groups. **(D)** GO enrichment map of 107 differentially expressed genes. **(E)** KEGG enrichment map of 107 differentially expressed genes. TRP, transient receptor potential; AML, acute myeloid leukemia; GO, Gene Ontology; KEGG, Kyoto Encyclopedia of Genes Genomes. **,<0.01; *** ,<0.001; and ****,<0.0001.

We calculated TESs for each AML patient using the ssGSEA algorithm. The correlations among the TRGs, clinicopathological characteristics, and TESs are exhibited in **Figure 1A**. Compared with AML patients with lower TESs, the expressions of TRPM1, TRPM2, TRPM5, and TRPM5 were relatively high; on the contrary, the expressions of TRPM6 and TRPM7 were relatively low (**Figure 1A**). We distinguished AML patients into the HTS group and LTS group. From **Figure 1B**, we can observe the significantly worse clinical outcomes of patients with higher TESs, while the prognosis of patients with lower TESs was better. Therefore, TES may be a driving factor for the malignant progression of AML. There were 107 differentially expressed TRGs identified by differential analysis (logFC > 1, p < 0.05), which could be reflected in the volcano map (**Figure 1C**). We carried out an enrichment analysis to explore the biological function of these differentially expressed TRGs. The GO analysis (**Figure 1D**) showed that these TRGs were significantly enriched in several immune-related pathways (neutrophil activation, neutrophil degranulation, neutrophil activation involved in immune response, neutrophil-mediated immunity, defense response to bacterium, defense response to fungus, negative regulation of immune system process, leukocyte migration, macrophage activation, and macrophage differentiation). KEGG analysis (**Figure 1E**) revealed that these TRGs were significantly enriched in some classical tumor-related pathways (Transcriptional misregulation in cancer, IL-17 signaling pathway, Arachidonic acid metabolism, Influenza A, C-type lectin receptor signaling pathway, Bladder cancer, Serotonergic synapse, Malaria, Shigellosis, and Melanoma).

### Establishment of transient receptor potential signature

**Figure 2:**
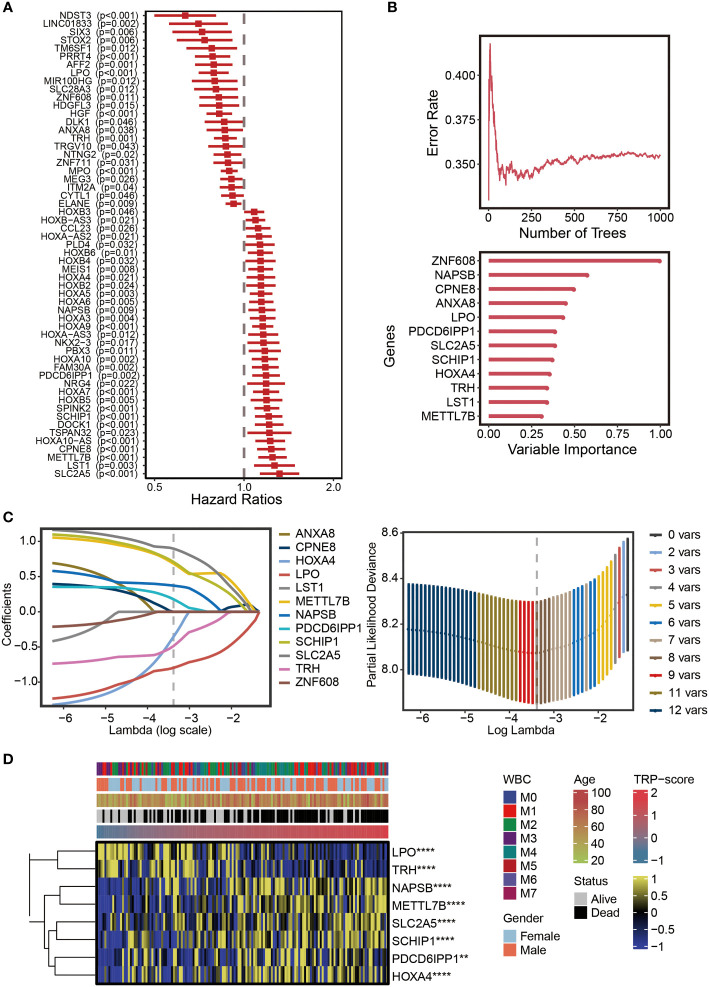
Establishment of TRP signature. **(A)** Forest plot for univariate Cox regression analysis of 57 prognostic TRP-related genes. **(B)** The distribution of error rates in Random Survival Forest model and the variable relative importance of 12 TRP-related genes (variable importance >0.3). **(C)** Lambda selection diagram for least absolute shrinkage and selection operator (Lasso) analysis. **(D)** The heatmap displaying the distribution of the eight TRP-related genes of the signature, clinicopathological characteristics, and TRP enrichment scores. Yellow represents high gene expression; blue represents low gene expression. TRP, transient receptor potential. **,<0.01; and ****,<0.0001.

The univariate Cox regression analysis was performed on the differentially expressed TRGs obtained above. The results revealed 57 prognostic TRGs (**Figure 2A**), including 24 potential tumor-protective factors (hazard ratio (HR) < 1) and 33 potential tumor-promoting factors (HR > 1). Thereafter, the distribution of error rates generated by the Random Survival Forest model is shown in **Figure 2B**, thus identifying the variable importance (variable importance >0.3, **Figure 2B**) of 12 TRGs (ZNF608, NAPSB, CPNE8, ANXA8, LPO, PDCD6IPP1, SLC2A5, SCHIP1, HOXA4, TRH, LST1, and METTL7B). Lasso analysis was used to construct the TRP signature, and the TRP score for 149 patients with AML was calculated. Ultimately, eight TRGs (ANXA8, CPNE8, HOXA4, LPO, LST1, METTL7B, NAPSB, PDCD6IPP1, SCHIP1, SLC2A5, TRH, and ZNF608) were obtained to establish the prognostic signature, and **Figure 2C** displays the lambda selection diagram. The heatmap displays the distribution of the eight TRGs of the signature, clinicopathological characteristics, and TRP score. It can be clearly observed that high expression of LPO and TRH may be associated with lower TRP scores, in contrast to high expression of NAPSB, METTL7B, SLC2A5, SCHIP1, PDCD6IPP1, and HOXA4, which was associated with higher TRP scores (**Figure 2D**).

### Efficacy of transient receptor potential signature

**Figure 3:**
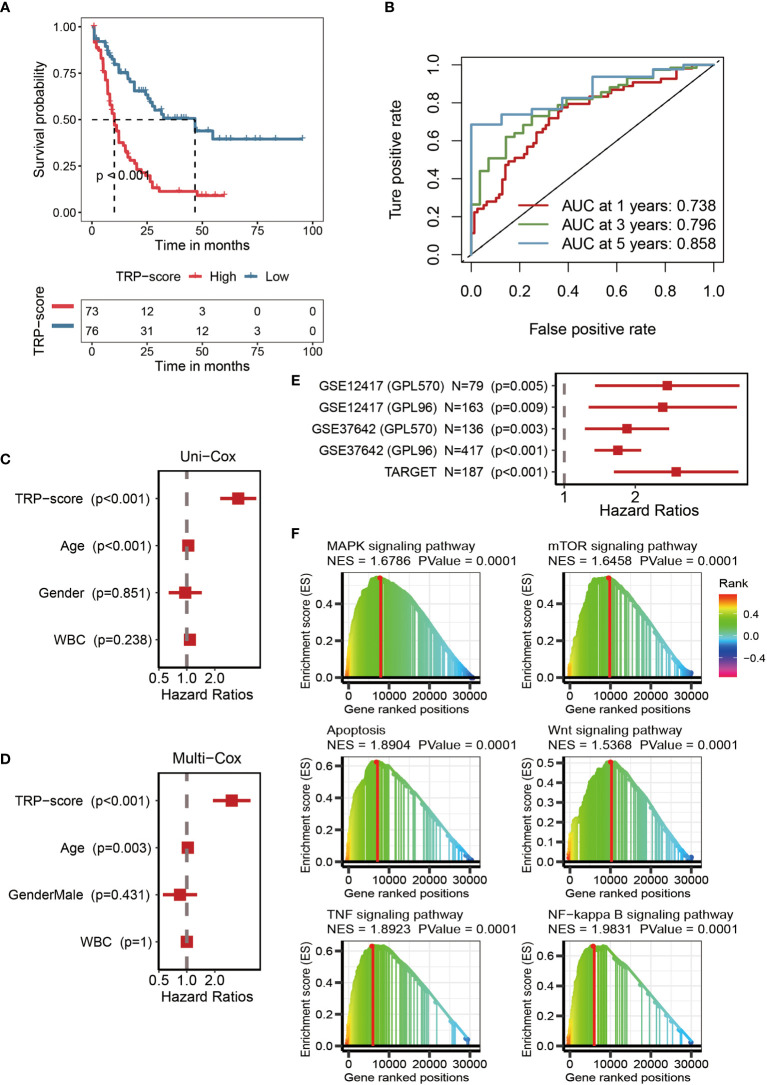
Efficacy of TRP signature. **(A)** Kaplan–Meier survival curve showing survival probability of high TRP score or low TRP score subgroups. The blue curve represents the group with lower TRP scores, and the red curve represents group with higher TRP scores. **(B)** The 1-year (0.738), 3-year (0.796), and 5-year (0.858) survival ROC curves predicted by the TRP signature. **(C)** The forest figure for univariate Cox regression analysis of TRP score and clinicopathological features. **(D)** The forest figure for multivariate Cox regression analysis of TRP score and clinicopathological features. **(E)** Univariate Cox regression analysis of the TRP signature in five external validation datasets (GSE12417-GPL570, GSE12417-GPL96, GSE37642-GPL570, GSE37642-GPL96, and TARGET). **(F)** GSEA showing cancer-related pathways positively regulated by TRP signature. TRP, transient receptor potential; ROC, receiver operating characteristic; GSEA, Gene Set Enrichment Analysis.

The optimal cutoff of TRP scores was set as a threshold value to distinguish AML patients into the HTS or LTS groups. The details of clinic information are listed in **Supplementary Table S1**. The survival curves showed significantly worse clinical outcomes of patients with higher TRP scores, while the prognosis of patients with lower TRP scores was better (**Figure 3A**). The area under the curve (AUCs) values of 1-year (AUC = 0.738), 3-year (AUC = 0.796), and 5-year (AUC = 0.858) survival ROC curves predicted by the TRP signature were all higher than 0.7, suggesting the efficiency of TRP signature in predicting prognosis for AML (**Figure 3B**). Furthermore, TRP signature was an independent prognostic factor for AML patients as demonstrated by univariate (**Figure 3C**) and multivariate (**Figure 3D**) Cox regression analyses. Finally, univariate Cox regression analysis was conducted on five external validation datasets (GSE12417-GPL570, GSE12417-GPL96, GSE37642-GPL570, GSE37642-GPL96, and TARGET), and the HRs of the five sets were all greater than 1, demonstrating the accuracy of the TRP signature that we constructed in prognostic prediction (**Figure 3E**, **Supplementary Figure S1**). Based on GSEA, six cancer-related pathways (MAPK signaling pathway, TOR signaling pathway, Apoptosis, Wnt signaling pathway, TNF signaling pathway, and NF-kappa B signaling pathway) were identified, which may be positively regulated by this signature, which provided insights for exploring the mechanism of AML (**Figure 3F**).

### Genomic mutation analysis for transient receptor potential signature

**Figure 4:**
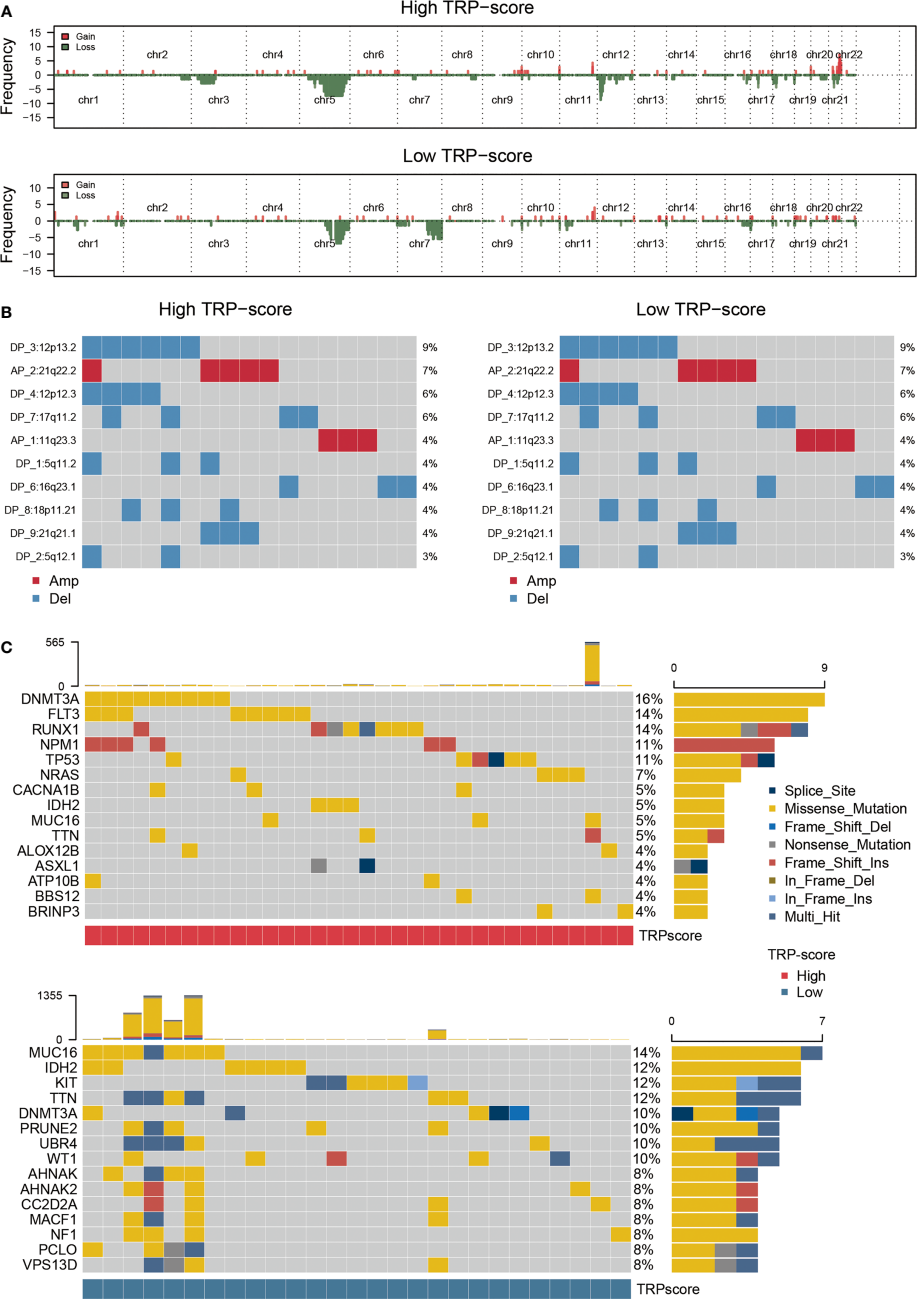
Genomic mutation analysis for TRP signature. **(A)** Genomic characterization landscape of groups with high TRP scores or low TRP scores. **(B)** The detailed amplificated or deleted CNV onco-plots of groups with high TRP scores or low TRP scores. **(C)** Waterfall plot of somatic mutations in AML between high and low TRP score groups. TRP, transient receptor potential; AML, acute myeloid leukemia; CNV, copy number variation.

We assessed the genomic characterization landscape of the HTS group or LTS group by the GISTIC algorithm, as shown in **Figure 4A**. Further, we plotted the detailed amplificated or deleted CNV onco-plots of the HTS and LTS groups (**Figure 4B**). From **Figure 4B**, we can observe that the results of the two subgroups were similar. DNMT3A, FLT3, RUNX1, NPM1, TP53, NRAS, CACNA1B, IDH2, MUC16, TTN, ALOX12B, ASXL1, ATP10B, BBS12, and BRINP3 were the top 15 genes with the highest mutation rate in AML patients with high TRP scores (**Figure 4C**). MUC16, IDH2, KIT, TTN, DNMT3A, PRUNE2, UBR4, WT1, AHNAK, AHNAK2, CC2D2A, MACF1, NF1, PCLO, and VPS13D were the top 15 genes with the highest mutation rate in AML patients with low TRP scores (**Figure 4C**). Thus, the mutation rates of DNMT3A, IDH2, MUC16, and TTN in the two subgroups were relatively high.

### Evaluation of immunological characteristics for transient receptor potential signature

**Figure 5:**
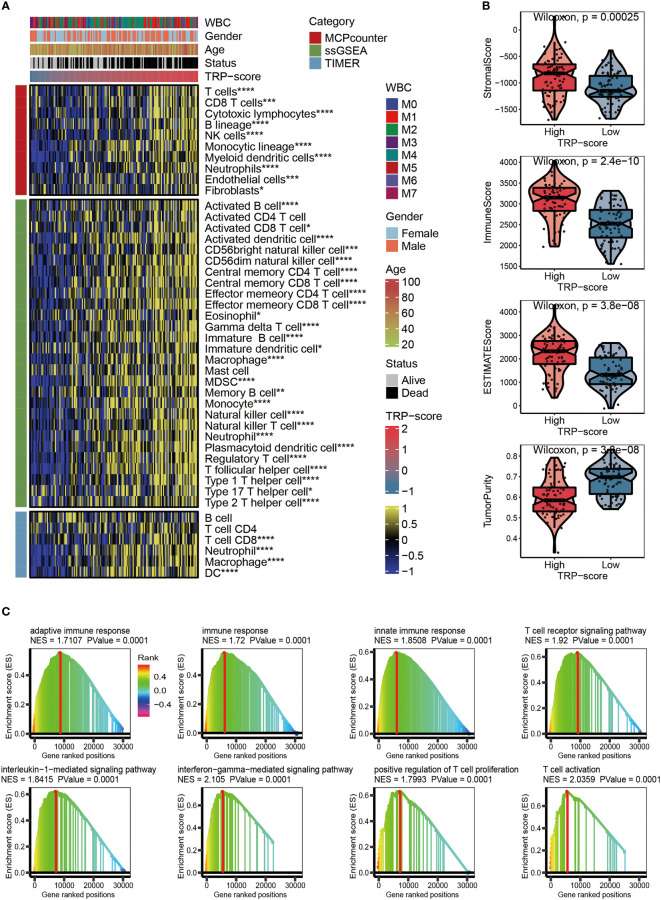
Evaluation of immunological characteristics for TRP signature. **(A)** Heatmap displaying the abundance of infiltrating immune cell populations with different TRP scores. **(B)** The violin chart comparing the differences between high and low TRP scores on stromal score, immune score, ESTIMATE score, and tumor purity. **(C)** GSVA for immune-related pathways positively regulated by TRP signature. TRP, transient receptor potential; GSVA, gene set variation analysis. *,<0.05; **,<0.01; ***,<0.001; and ****,<0.0001.

After analysis based on MCPcounter, ssGSEA, and TIMER algorithms, the abundance of infiltrating immune cell populations with different TRP scores was displayed in the heatmap (**Figure 5A**). From a general view, the level of infiltrating immune cell populations (**Figure 5A**), stromal score (**Figure 5B**), immune score (**Figure 5B**), and ESTIMATE score (**Figure 5B**) increased as the TRP scores increased. Nevertheless, AML patients with lower TRP scores exhibited more tumor purity (**Figure 5B**). As for gene set variation analysis (GSVA), we focused on immune-related pathways positively regulated by TRP signature. The results showed that the TRP signature may be associated with adaptive immune response, immune response, innate immune response, T-cell receptor signaling pathway, interleukin-1-mediated signaling pathway, interferon-gamma-mediated signaling pathway, positive regulation of T cell proliferation, and T-cell activation (**Figure 5C**).

### Immunotherapy and chemotherapy of transient receptor potential signature

**Figure 6:**
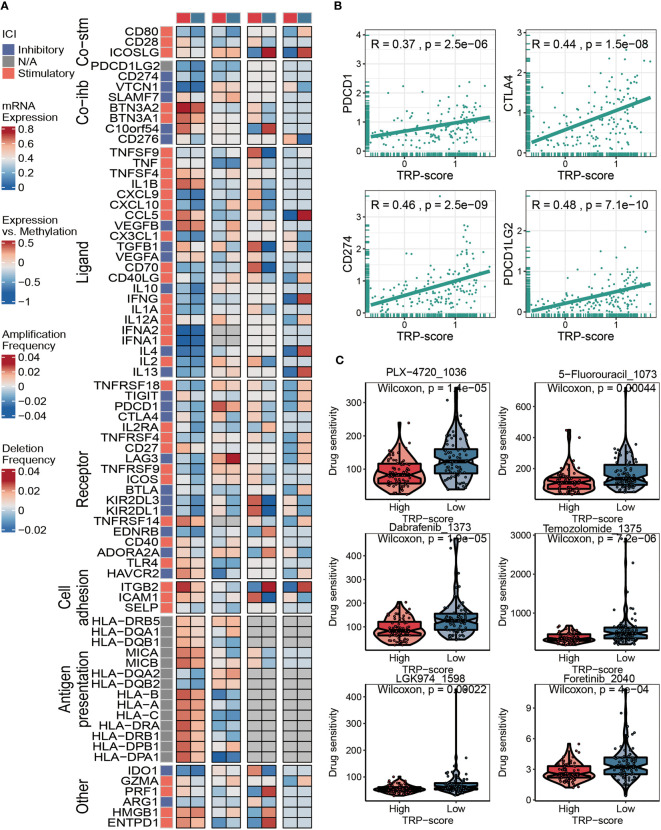
Immunotherapy and chemotherapy of TRP signature for AML. **(A)** Correlation of TRP score with seven immunomodulators in AML. **(B)** Correlation between expression of four immune checkpoints and TRP scores. **(C)** Box plots of estimated IC50 for six chemotherapeutic agents in the high or low TRP score groups. TRP, transient receptor potential; AML, acute myeloid leukemia.

Considering that immunomodulators (IMs) play a critical role in tumor immunotherapy, we compared the correlation between immunomodulator levels (Co-stm, Co-ihb, Ligand, Receptor, Cell adhesion, Antigen presentation, and Other) and the prognostic TRP signature (**Figure 6A**). To further evaluate the relationship between TRP score and immunotherapy, we calculated the correlation between the TRP scores and the expression level of four classical immune checkpoints, and we found that the score was correlated with PDCD1 (R = 0.37, p = 2.5 × e^−6^), CTLA4 (R = 0.44, p = 1.5 × e^−8^), CD274 (R = 0.46, p = 2.5 × e^−9^), and PDCD1LG2 (R = 0.48, p = 7.1 × e^−10^), which can provide an important reference for the immunotherapy of AML (**Figure 6B**). The IC50 values of six chemotherapeutics (PLX-4720, 5-Fluorouracil-1073, Dabrafenib-1373, Temozolomide-1375, LGK974-1598, and Foretinib-2040) were contrasted using violin figures, and our data revealed that the IC50 values of the chemotherapeutics mentioned above were lower in the HTS group than in the LTS group, suggesting that patients with higher TRP scores were more likely to benefit from these six chemotherapeutics (**Figure 6C**).

### *In vitro* assays

**Figure 7:**
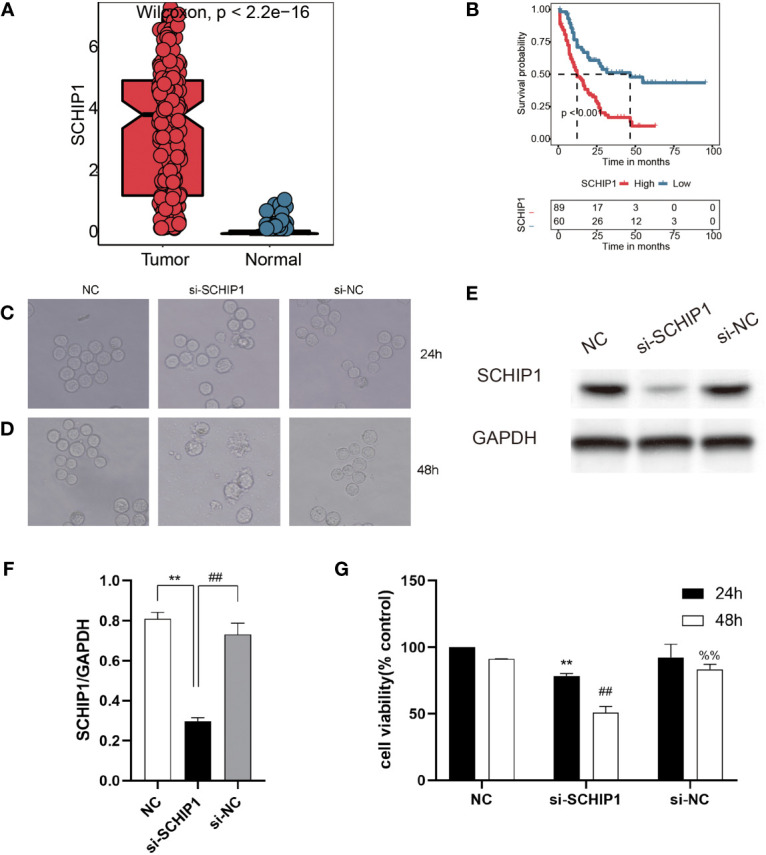
*In vitro* cyto-functional experiments for SCHIP1 of AML. **(A, B)** The expression of SCHIP1 in TCGA between normal and tumor. **(B)** The prognosis of SCHIP1 in TCGA. Cell morphology after transfection at 24 h **(C** or **D)**. NC denotes blank control group, si-SCHIP1 denotes knockdown SCHIP1 group, and si-NC denotes control group. **(E, F)** Western blotting assays verifying the transfection efficiency. **(G)** CCK8 assays comparing the survival rate of different groups of cells. AML, acute myeloid leukemia; TCGA, The Cancer Genome Atlas; CCK8, Cell Counting Kit-8. **,<0.01; si-SCHIP1 vs NC for SCHIP1/GAPDH and 24h cell viability; ##, <0.01; si-NC vs si-SCHIP1 for SCHIP1/GAPDH and 48h cell viability; %%, <0.01; si-NC vs si-SCHIP1 for 48h cell viability.

To verify the effect of TRP score *in vitro*, we selected SCHIP1, as it represents genes of TRP score in further work. First, our results showed that there was a significant difference in the expression of SCHIP1 between the tumor and normal, and the SCHIP1 also had a poor prognosis in TCGA-AML cohort (**Figures 7A, B**). Then, after cell transfection, we observed the cell morphology under the microscope (**Figure 7C**). As shown in **Figure 7D**, 24 h after transfection, the cells in the NC group had regular shape and uniform size, and there was no significant difference between the si-NC and NC groups, while the cells in the si-SCHIP1 group had heterogeneous size and irregular shape, and some cells showed apoptosis. Forty-eight hours after transfection, the cell morphology of the NC group and si-NC group was regular, and there was no significant difference between the two groups, while the si-SCHIP1 group showed significant apoptosis. We used Western blotting assays to detect the knockout efficiency of SCHIP1 gene, and the results showed that compared with the control group, SCHIP1 gene in the si-SCHIP1 group was significantly knocked down after cell transfection (**Figures 7E, F**). We used CCK8 assay to test the cell viability of each group (**Figure 7G**). We found that after 24 h, the cell viability of the si-SCHIP1 group was significantly lower than that of the control group (p < 0.01). After 48 h, the cell viability in the si-SCHIP1 group was also significantly decreased compared with the control group (p < 0.01), while the cell viability in the si-NC group was significantly increased compared with the si-SCHIP1 group (p < 0.01).

## Discussion

Since the discovery of AML, a great deal of research has been carried out on its etiology, development, and treatment. With the development of technology and in-depth research, many important prognostic factors have been found, such as age, chromosome typing, genotyping, and initial and white blood cell count, and patients are grouped according to these prognostic factors to guide diagnosis and treatment (47). However, due to the limitation of traditional clinicopathological features, the clinical prognosis of patients with AML is still highly heterogeneous. According to the European LeukemiaNet (ELN) risk classification system, about half of patients are classified into the intermediate risk group (48). AML is one of the most common malignant diseases of the circulatory system. Different types of AM L may have different clinical manifestations and prognoses. In conjunction with this change, there is a growing acceptance of early risk stratification for AML to guide further treatment. Clear risk stratification of AML is the prerequisite for subsequent correct diagnosis and treatment. In this study, we established risk stratification based on TRP scores. The survival curves showed significantly worse clinical outcomes for patients with higher TRP scores, while the prognosis of patients with lower TRP scores was better. The AUC values of 1-year (AUC = 0.738), 3-year (AUC = 0.796), and 5-year (AUC = 0.858) survival ROC curves predicted by the TRP scores were all higher than 0.7, suggesting the efficiency of TRP signature in predicting prognosis for AML. Furthermore, the TRP score was an independent prognostic factor for AML patients demonstrated by univariate and multivariate Cox regression analyses. The accuracy of the TRP score we constructed in prognostic prediction was recognized by five external validation datasets. In conclusion, the TRP score system may be a novel and reliable stratification system for AML.

As a rapidly developing interdisciplinary, bioinformatics uses computer science and mathematics to drive the development of biology. Traditional biological studies and clinical studies are often limited, and the selection of research objectives is often through theoretical speculation or literature support. However, bioinformatics research is more macroscopic. Based on the massive data obtained at the gene level or protein level, high-throughput sequencing technology and other technologies are used to screen out some more potential research targets, which provides possible directions for research. TCGA database is a joint project of the National Cancer Institute (NCI) and the National Human Genome Research Institute (NHGRI) so as to help researchers better understand cancer and promote related cancer prevention, diagnosis, and treatment progress (4). The GTEx database stores a large number of human normal tissue sequencing samples, which can be used to analyze the genetic differences between tumor samples and normal samples (49). The GEO database is a project of the National Center for Biotechnology Information (NCBI), which stores data mainly from microarray or sequencing data uploaded by various research institutions and individuals. GPL refers to the type of sequencing platform used for sequencing data or gene chip data, and GSE refers to the sequencing data dataset of a series of samples (50). In this study, we carried out a comprehensive bioinformatics analysis based on data from TCGA-LAML dataset in the UCSC Xena platform, GTEx, GEO, and TARGET datasets. We also used a number of algorithms (KEGG, GO, ORA, GSEA, GISTIC, ESTIMATE, TIMER, MCPcounter, ssGSEA, and pRRophetic) to assess functional enrichment pathways, somatic mutations, immune characteristics, and drug sensitivity in AML. Bioinformatics analysis contributed to our results.

In addition to using a large number of bioinformatics tools for analysis, this study also selected a gene, SCHIP1, for the wet experiment, which is also a highlight of this study. SCHIP1 is located at chromosome 3q25 and is a relatively unusual protein initially discovered through interactions with the tumor inhibitor Merlin/NF2 in the mouse brain, and it is a new member of the Hippo pathway (51, 52). SCHIP1 plays different roles in many diseases. SCHIP1 has a variety of functions and plays an important role in the organization of Langhock during early brain development and adulthood, and SCHIP1 is also a cytoplasmic chaperone for cortical cytoskeletal tonic proteins (53). Studies have shown that SCHIP1 plays an important role in proteinuria (54). SCHIP1 also promotes the development and progression of several tumors, including adrenal tumors, acute lymphoblastic leukemia, renal cell carcinoma, and colorectal cancer (55–58). Zhang et al. proposed that IQCJ-Schip1-AS1 could affect the proliferation of colorectal cancer cells through the pathways of cell cycling, DNA replication, and p53 (58). In addition, SCHIP1 is an NF2/Merlin interacting protein in *Drosophila*, and its coiled-coil domain interacts with NF2/Merlin to influence the Hippo pathway (52). After the knockdown of SCHIP1, we found that the apoptosis of AML cells increased and the cell growth rate slowed down, which indicated that SCHIP1 may be a malignant promoter of AML.

There are limitations to the study. First, we constructed and validated the risk prognostic model by retrospectively studying the public database, while more prospective studies are needed for clinical practicability. Second, due to the older AML project data in TCGA database, the lack of clinical information is serious. At the same time, there are few AML data with rich clinical information, and the lack of clinically relevant data is inevitable in this study. Finally, different from solid tumors, which usually detect differential genes by comparing tumor tissues with adjacent tissues, hematological tumors are inevitably affected by other external factors due to the lack of normal bone marrow cells in the samples themselves.

## Conclusions

This study determined a risk stratification system based on TRP score through detailed bioinformatics analysis and initially confirmed that SCHIP1 is the oncogene of AML.

## Data availability statement

The original contributions presented in the study are included in the article/supplementary material. Further inquiries can be directed to the corresponding authors.

## Author contributions

TD and YS conceived, designed, and supervised the study. JH performed the data analysis and drafted the manuscript. All authors contributed to the article and approved the submitted version.

## Supplementary material

Supplementary Figure 1 Kaplan–Meier curves for five external validation data sets.

## Glossary

**Table:**

AML	acute myeloid leukemia
GTEx	Genome-Tissue Expression
TRG	TRP-related gene
ssGSEA	Single-Sample Gene Set Enrichment Analysis
TES	TRP enrichment score
DEG	differentially expressed gene
Lasso	least absolute shrinkage and selection operator
ROC	receiver operating characteristic
GISTIC	Genomic Identification of Significant Targets in Cancer
KEGG	Kyoto Encyclopedia of Genes Genomes
GO	Gene Ontology
ORA	over-representation analysis
GSEA	Gene Set Enrichment Analysis
GDSC	Genomics of Drug Sensitivity in Cancer
CR	complete remission
TCGA	The Cancer Genome Atlas
TRP	transient receptor potential
TARGET	Therapeutically Applicable Research to Generate Effective Treatments
RMA	Robust Multichip Average
FPKM	fragments per kilobase of transcript per million fragments mapped
TPM	transcripts per kilobase million
HTS	high TRP score
LTS	low TRP score
OS	overall survival
CNV	copy number variation
ESTIMATE	Estimation of Stromal and Immune cells in Malignant Tumor tissues using Expression
TIMER	Tumor Immune Estimation Resource
RPMI	Roswell Park Memorial Institute
FBS	fetal bovine serum
PMSF	phenylmethylsulfonyl fluoride
PVDF	olyvinylidene difluoride
SCHIP1,	Schwannomin-Interacting Protein 1

## References

[1] Döhner H Estey E Grimwade D Amadori S Appelbaum FR Büchner T. Diagnosis and management of AML in adults: 2017 ELN recommendations from an international expert panel. Blood (2017) 4:424–47. doi: 10.1182/blood-2016-08-733196 PMC5291965 27895058

[2] Freireich EJ Wiernik PH Steensma DP. The leukemias: a half-century of discovery. J Clin Oncol (2014) 32:3463–9. doi: 10.1200/jco.2014.57.1034 25185093

[3] Ouzounis CA Valencia A. Early bioinformatics: the birth of a discipline–a personal view. Bioinformatics (2003) 19:2176–90. doi: 10.1093/bioinformatics/btg309 14630646

[4] Wang Z Jensen MA Zenklusen JC. A practical guide to the cancer genome atlas (TCGA). Methods Mol Biol (2016) 1418:111–41. doi: 10.1007/978-1-4939-3578-9_6 27008012

[5] Himmel NJ Cox DN. Transient receptor potential channels: current perspectives on evolution, structure, function and nomenclature. Proc Biol Sci (2020) 287:20201309. doi: 10.1098/rspb.2020.1309 32842926 PMC7482286

[6] Cao E. Structural mechanisms of transient receptor potential ion channels. J Gen Physiol (2020) 152:e201811998. doi: 10.1085/jgp.201811998 31972006 PMC7054860

[7] Li H. TRP channel classification. Adv Exp Med Biol (2017) 976:1–8. doi: 10.1007/978-94-024-1088-4_1 28508308

[8] Billeter AT Hellmann JL Bhatnagar A Polk HC Jr. Transient receptor potential ion channels: powerful regulators of cell function. Ann Surg (2014) 259:229–35. doi: 10.1097/SLA.0b013e3182a6359c 23989052

[9] Tjong YW Yao X. Methods for evaluation of vascular endothelial cell function with transient receptor potential (TRP) channel drugs. Methods Mol Biol (2018) 1722:195–210. doi: 10.1007/978-1-4939-7553-2_13 29264807

[10] Everaerts W Vriens J Owsianik G Appendino G Voets T De Ridder D. Functional characterization of transient receptor potential channels in mouse urothelial cells. Am J Physiol Renal Physiol (2010) 3:F692–701. doi: 10.1152/ajprenal.00599.2009 PMC2838597 20015940

[11] Holzer P. Transient receptor potential (TRP) channels as drug targets for diseases of the digestive system. Pharmacol Ther (2011) 131:142–70. doi: 10.1016/j.pharmthera.2011.03.006 PMC3107431 21420431

[12] Stokłosa P Borgström A Kappel S Peinelt C. TRP channels in digestive tract cancers. Int J Mol Sci (2020) 21:1877. doi: 10.3390/ijms21051877 32182937 PMC7084354

[13] Julius D. TRP channels and pain. Annu Rev Cell Dev Biol (2013) 29:355–84. doi: 10.1146/annurev-cellbio-101011-155833 24099085

[14] Basso L Altier C. Transient receptor potential channels in neuropathic pain. Curr Opin Pharmacol (2017) 32:9–15. doi: 10.1016/j.coph.2016.10.002 27835802

[15] Vangeel L Voets T. Transient receptor potential channels and calcium signaling. Cold Spring Harb Perspect Biol (2019) 11:a035048. doi: 10.1101/cshperspect.a035048 30910771 PMC6546042

[16] Chen SJ Bao L Keefer K Shanmughapriya S Chen L Lee J. Transient receptor potential ion channel TRPM2 promotes AML proliferation and survival through modulation of mitochondrial function, ROS, and autophagy. Cell Death Dis (2020) 4:247. doi: 10.1038/s41419-020-2454-8 PMC7170900 32312983

[17] Wang F Wu P Gong S Chen Y Gao J Wang S. Aberrant TRPM4 expression in MLL-rearranged acute myeloid leukemia and its blockade induces cell cycle arrest *via* AKT/GLI1/Cyclin D1 pathway. Cell Signal (2020) 72:109643. doi: 10.1016/j.cellsig.2020.109643 32320859

[18] Goldman MJ Craft B Hastie M Repečka K McDade F Kamath A. Visualizing and interpreting cancer genomics data *via* the xena platform. Nat Biotechnol (2020) 38:675–8. doi: 10.1038/s41587-020-0546-8 PMC7386072 32444850

[19] Human genomics. the genotype-tissue expression (GTEx) pilot analysis: multitissue gene regulation in humans. Science (2015) 348:648–60. doi: 10.1126/science.1262110 PMC4547484 25954001

[20] Bolouri H Farrar JE Triche T Jr Ries RE Lim EL Alonzo TA. The molecular landscape of pediatric acute myeloid leukemia reveals recurrent structural alterations and age-specific mutational interactions. Nat Med (2018) 1:103–12. doi: 10.1038/nm.4439 PMC5907936 29227476

[21] Dalma-Weiszhausz DD Warrington J Tanimoto EY Miyada CG. The affymetrix GeneChip platform: an overview. Methods Enzymol (2006) 410:3–28. doi: 10.1016/s0076-6879(06)10001-4 16938544

[22] Wolber PK Collins PJ Lucas AB De Witte A Shannon KW. The agilent in situ-synthesized microarray platform. Methods Enzymol (2006) 410:28–57. doi: 10.1016/s0076-6879(06)10002-6 16938545

[23] Katz S Irizarry RA Lin X Tripputi M Porter MW. A summarization approach for affymetrix GeneChip data using a reference training set from a large, biologically diverse database. BMC Bioinf (2006) 7:464. doi: 10.1186/1471-2105-7-464 PMC1624855 17059591

[24] Zhao S Ye Z Stanton R. Misuse of RPKM or TPM normalization when comparing across samples and sequencing protocols. Rna (2020) 26:903–9. doi: 10.1261/rna.074922.120 PMC7373998 32284352

[25] Samanta A Hughes TET Moiseenkova-Bell VY. Transient receptor potential (TRP) channels. Subcell Biochem (2018) 87:141–65. doi: 10.1007/978-981-10-7757-9_6 PMC6038138 29464560

[26] Hänzelmann S Castelo R Guinney J. GSVA: gene set variation analysis for microarray and RNA-seq data. BMC Bioinf (2013) 14:7. doi: 10.1186/1471-2105-14-7 PMC3618321 23323831

[27] Zhang LP Ren H Du YX Wang CF. Prognostic value of the preoperative fibrinogen-to-albumin ratio in pancreatic ductal adenocarcinoma patients undergoing R0 resection. World J Gastroenterol (2020) 26:7382–404. doi: 10.3748/wjg.v26.i46.7382 PMC7739158 33362391

[28] Ritchie ME Phipson B Wu D Hu Y Law CW Shi W. Limma powers differential expression analyses for RNA-sequencing and microarray studies. Nucleic Acids Res (2015) 7:e47. doi: 10.1093/nar/gkv007 PMC4402510 25605792

[29] van Dijk PC Jager KJ Zwinderman AH Zoccali C Dekker FW. The analysis of survival data in nephrology: basic concepts and methods of cox regression. Kidney Int (2008) 74:705–9. doi: 10.1038/ki.2008.294 18596734

[30] Taylor JM. Random survival forests. J Thorac Oncol (2011) 6:1974–5. doi: 10.1097/JTO.0b013e318233d835 22088987

[31] Tibshirani R. The lasso method for variable selection in the cox model. Stat Med (1997) 16:385–95. doi: 10.1002/(sici)1097-0258(19970228)16:4<385::aid-sim380>3.0.co;2-3 9044528

[32] Ranstam J Cook JA. Kaplan-Meier Curve. Br J Surg (2017) 104:442. doi: 10.1002/bjs.10238 28199017

[33] Gao J Aksoy BA Dogrusoz U Dresdner G Gross B Sumer SO. Integrative analysis of complex cancer genomics and clinical profiles using the cBioPortal. Sci Signal (2013) 269:pl1. doi: 10.1126/scisignal.2004088 PMC4160307 23550210

[34] Chabanais J Labrousse F Chaunavel A Germot A Maftah A. POFUT1 as a promising novel biomarker of colorectal cancer. Cancers (Basel) (2018) 10:411. doi: 10.3390/cancers10110411 30380753 PMC6266312

[35] Mermel CH Schumacher SE Hill B Meyerson ML Beroukhim R Getz G. GISTIC2.0 facilitates sensitive and confident localization of the targets of focal somatic copy-number alteration in human cancers. Genome Biol (2011) 4:R41. doi: 10.1186/gb-2011-12-4-r41 PMC3218867 21527027

[36] Yoshihara K Shahmoradgoli M Martínez E Vegesna R Kim H Torres-Garcia W. Inferring tumour purity and stromal and immune cell admixture from expression data. Nat Commun (2013) 4:2612. doi: 10.1038/ncomms3612 24113773 PMC3826632

[37] Li T Fan J Wang B Traugh N Chen Q Liu JS. TIMER: A web server for comprehensive analysis of tumor-infiltrating immune cells. Cancer Res (2017) 21:e108–10. doi: 10.1158/0008-5472.Can-17-0307 PMC6042652 29092952

[38] Becht E Giraldo NA Lacroix L Buttard B Elarouci N Petitprez F. Estimating the population abundance of tissue-infiltrating immune and stromal cell populations using gene expression. Genome Biol (2016) 1:218. doi: 10.1186/s13059-016-1070-5 PMC5073889 27765066

[39] Xiao B Liu L Li A Xiang C Wang P Li H. Identification and verification of immune-related gene prognostic signature based on ssGSEA for osteosarcoma. Front Oncol (2020) 10:607622. doi: 10.3389/fonc.2020.607622 33384961 PMC7771722

[40] Sacks D Baxter B Campbell BCV Carpenter JS Cognard C Dippel D. Multisociety consensus quality improvement revised consensus statement for endovascular therapy of acute ischemic stroke. Int J Stroke (2018) 6:612–32. doi: 10.1177/1747493018778713 29786478

[41] Liberzon A Birger C Thorvaldsdóttir H Ghandi M Mesirov JP Tamayo P. The molecular signatures database (MSigDB) hallmark gene set collection. Cell Syst (2015) 6:417–25. doi: 10.1016/j.cels.2015.12.004 PMC4707969 26771021

[42] Yu G Wang LG Han Y He QY. clusterProfiler: an r package for comparing biological themes among gene clusters. Omics (2012) 16:284–7. doi: 10.1089/omi.2011.0118 PMC3339379 22455463

[43] Subramanian A Tamayo P Mootha VK Mukherjee S Ebert BL Gillette MA. Gene set enrichment analysis: a knowledge-based approach for interpreting genome-wide expression profiles. Proc Natl Acad Sci USA (2005) 43:15545–50. doi: 10.1073/pnas.0506580102 PMC1239896 16199517

[44] Yang W Soares J Greninger P Edelman EJ Lightfoot H Forbes S. Genomics of drug sensitivity in cancer (GDSC): A resource for therapeutic biomarker discovery in cancer cells. Nucleic Acids Res (2013) Database issue:D955–61. doi: 10.1093/nar/gks1111 PMC3531057 23180760

[45] Geeleher P Cox NJ Huang RS. Clinical drug response can be predicted using baseline gene expression levels and *in vitro* drug sensitivity in cell lines. Genome Biol (2014) 15:R47. doi: 10.1186/gb-2014-15-3-r47 24580837 PMC4054092

[46] Geeleher P Cox N Huang RS. pRRophetic: an r package for prediction of clinical chemotherapeutic response from tumor gene expression levels. PLoS One (2014) 9:e107468. doi: 10.1371/journal.pone.0107468 25229481 PMC4167990

[47] Papaemmanuil E Gerstung M Bullinger L Gaidzik VI Paschka P Roberts ND. Genomic classification and prognosis in acute myeloid leukemia. N Engl J Med (2016) 23:2209–21. doi: 10.1056/NEJMoa1516192 PMC4979995 27276561

[48] Döhner H Estey EH Amadori S Appelbaum FR Büchner T Burnett AK. Diagnosis and management of acute myeloid leukemia in adults: recommendations from an international expert panel, on behalf of the European LeukemiaNet. Blood (2010) 3:453–74. doi: 10.1182/blood-2009-07-235358 19880497

[49] GTEx Consortium. The genotype-tissue expression (GTEx) project. Nat Genet (2013) 6:580–5. doi: 10.1038/ng.2653 PMC4010069 23715323

[50] Barrett T Wilhite SE Ledoux P Evangelista C Kim IF Tomashevsky M. NCBI GEO: archive for functional genomics data sets–update. Nucleic Acids Res (2013) Database issue:D991–5. doi: 10.1093/nar/gks1193 PMC3531084 23193258

[51] Goutebroze L Brault E Muchardt C Camonis J Thomas G. Cloning and characterization of SCHIP-1, a novel protein interacting specifically with spliced isoforms and naturally occurring mutant NF2 proteins. Mol Cell Biol (2000) 20:1699–712. doi: 10.1128/mcb.20.5.1699-1712.2000 PMC85353 10669747

[52] Chung HL Choi KW. Schip1, a new upstream regulator of hippo signaling. Cell Cycle (2016) 15:2097–8. doi: 10.1080/15384101.2016.1191252 PMC4993537 27246165

[53] Klingler E Martin PM Garcia M Moreau-Fauvarque C Falk J Chareyre F. The cytoskeleton-associated protein SCHIP1 is involved in axon guidance, and is required for piriform cortex and anterior commissure development. Development (2015) 11:2026–36. doi: 10.1242/dev.119248 25953347

[54] Perisic L Rodriguez PQ Hultenby K Sun Y Lal M Betsholtz C. Schip1 is a novel podocyte foot process protein that mediates actin cytoskeleton rearrangements and forms a complex with Nherf2 and ezrin. PLoS One (2015) 3:e0122067. doi: 10.1371/journal.pone.0122067 PMC4373682 25807495

[55] Suarez-Merino B Hubank M Revesz T Harkness W Hayward R Thompson D. Microarray analysis of pediatric ependymoma identifies a cluster of 112 candidate genes including four transcripts at 22q12.1-q13.3. Neuro Oncol (2005) 1:20–31. doi: 10.1215/s1152851704000596 PMC1871622 15701279

[56] Leung W Neale G Behm F Iyengar R Finkelstein D Kastan MB. Deficient innate immunity, thymopoiesis, and gene expression response to radiation in survivors of childhood acute lymphoblastic leukemia. Cancer Epidemiol (2010) 3:303–8. doi: 10.1016/j.canep.2010.03.008 PMC2874127 20413363

[57] Fisher KE Yin-Goen Q Alexis D Sirintrapun JS Harrison W Benjamin Isett R. Gene expression profiling of clear cell papillary renal cell carcinoma: Comparison with clear cell renal cell carcinoma and papillary renal cell carcinoma. Mod Pathol (2014) 2:222–30. doi: 10.1038/modpathol.2013.140 23887297

[58] Zhang J Bian Z Jin G Liu Y Li M Yao S. Long non-coding RNA IQCJ-SCHIP1 antisense RNA 1 is downregulated in colorectal cancer and inhibits cell proliferation. Ann Transl Med (2019) 9:198. doi: 10.21037/atm.2019.04.21 PMC6545302 31205916

