# Supplementary figures and images for: A transient receptor potential channel-related model based on machine learning for evaluating tumor microenvironment and immunotherapeutic strategies in acute myeloid leukemia
Source: Front Immunol. 2022 Dec 16;13:1040661. doi: 10.3389/fimmu.2022.1040661 (PMC9800424; doi:10.3389/fimmu.2022.1040661)

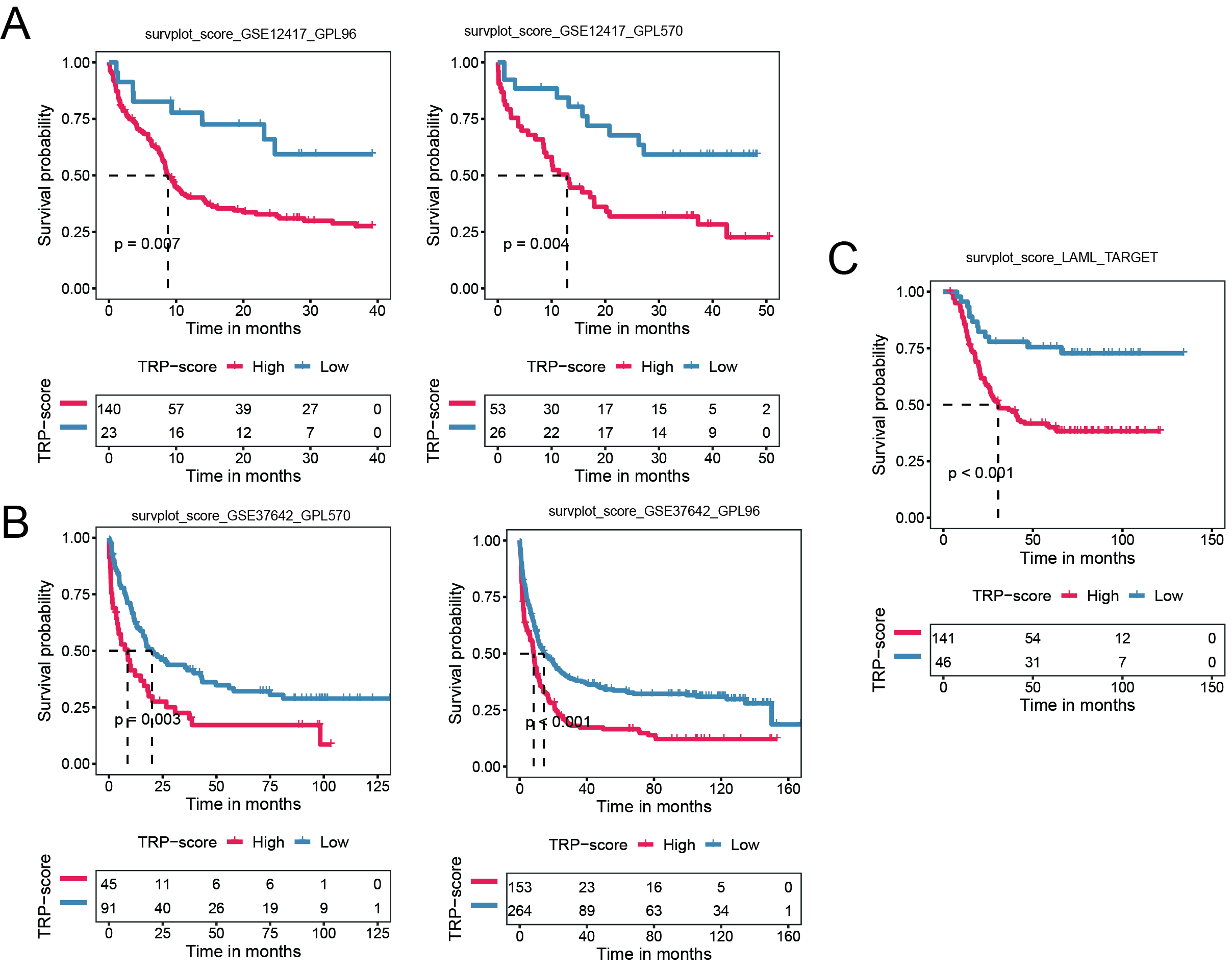

Supplement: Supplementary Figure 1 — Kaplan–Meier curves for five external validation data sets. [file Image_1.tif]
